# Commentary: GPR160 De-Orphanization Reveals Critical Roles in Neuropathic Pain in Rodents (Finally, a Receptor for CART Peptide)

**DOI:** 10.3389/adar.2021.10012

**Published:** 2021-10-07

**Authors:** Martin O. Job, Michael J. Kuhar

**Affiliations:** ^1^ Department of Biomedical Sciences, Cooper Medical School of Rowan University, Camden, NJ, United States; ^2^ Yerkes National Primate Research Center, Emory University, Atlanta, GA, United States

**Keywords:** GPR160, orphan receptor, CART peptide receptor, cocaine-and-amphetamine regulated transcript, GPCR, neurotransmitter

This commentary focuses on recent publications identifying a likely Cocaine-and-Amphetamine Regulated Transcript (CART) peptide (CARTp) receptor, namely the recently de-orphanized GPR160, some 26 years after the discovery of the CART mRNA. These publications are Yosten et al. [1], and Haddock et al. [2].

## Background

The discovery of the CART transcript and peptides implicated CARTp as a neurotransmitter involved in the action of psychostimulants and neuroendocrine regulation [3, 4]. But it became evident that it was involved in many additional physiological processes including body weight and feeding, energy expenditure, physical activity, body temperature, endocrine regulation, drug abuse and reward, pain, stress, hypertension, anxiety and depression, recovery from stroke, and possibly others. There are many reviews and other citations describing the evidence for these effects [5–30]. The general belief was and is that CARTp is an important peptide neurotransmitter. Several peptides derived from proCART are likely active [10, 14, 31].

Following the exciting discoveries of the peptide’s involvement in drug abuse [4, 7, 10, 11, 17, 30] and other processes, a critical need was for the identification and cloning of a CARTp receptor. There was evidence that a receptor existed, and it is briefly as follows. Responses to injections of CARTp were dose-responsive and the active peptide had structural requirements [14, 18, 22, 27, 30–40]. Injections of CARTp increased levels of second messengers, a common post receptor event [20, 21, 25, 28, 41–43]. Radiolabeled CARTp showed displaceable binding to neuronal cultured cells membranes, although and unusually so, not to adult brain tissue membranes [20, 27, 32, 42]. CARTp binding was altered by Gpp (nh)p - a G-protein binding ligand, and CARTp enhanced the binding of (35)S-GTP gamma S; these and other studies suggested that the receptor was a GPCR coupled to Gi/o [19–21, 28, 43, 44]. See also the reviews cited at the end of the last paragraph.

## Recent Discoveries

Given the existing evidence that a CARTp receptor is a GPCR, a reasonable approach to searching for a CART peptide receptor is to examine GPCR orphan receptors, receptors for which there are no known neurotransmitter ligands [29]. In studies of neuropathic pain, Yosten et al [1] found that an orphan receptor, GPR160 played a significant role in neuropathic pain and spinal cord. GPR160 was increased in spinal cord after traumatic nerve injury. Also, inhibition of GRR160 in the spinal cord prevented and reversed neuropathic pain but had no effect on normal pain. They then examined the connection between CARTp and GPR160 using antibodies (ab) and short interfering RNAs (si).

In KATO cancer cells, CARTp-induced cFOS expression and this was blocked by prior depletion of GPR160 using an si-GPR160. In PC12 cells expressing GPR160, CARTp stimulated ERK phosphorylation, but prior treatment with si-GPR160 reduced the effect. Also, CARTp co-immunoprecipitated with GPR160 protein indicating the likelihood of a physical interaction *in vivo*. Another finding was that injection of CARTab mimicked the effects of GPR160 inhibition. A CARTp-induced mechanohypersensitivity was dependent on GPR160. CARTp induced phosphorylation of ERK but this was attenuated with coinjection of GRP160ab. These data showed a functional and physical connection between CARTp and GPR160, at least in neuropathic pain.

Haddock et al [2] studied the CARTp receptor problem in the context of food and water intake. Injection of CARTp into the fourth ventricle reduced food and water intake, and this was prevented by immuno-neutralization of GPR160, again a connection between CARTp and GPR160. A hypothesis of circuitry and cellular localizations was made to provide a possible mechanism for these observations.

## Discussion

These findings strongly link the effects of CARTp to binding to an orphan receptor, GPR160. This initial identification of a CARTp receptor is a welcome observation after so many years of searching. Additional studies and confirmation of these findings are needed.

Could there be other CARTp receptors? It seems likely since most neurotransmitters have multiple receptors, and as noted above and elsewhere [45], there are several slightly different CARTps in different species and organs. In the recent studies discussed here, CARTp and GPR160 are implicated in neuropathic pain and food and water intake. Other receptors may be involved with other functions of CARTps.

This discovery will facilitate further research and understanding of the CARTp system in brain and periphery. It will also facilitate screening for small molecule agonists and antagonists, of which some mention has been made [20]. This will be very helpful in further studies of the functions of CARTps and for identifying therapeutic compounds based on CART.

## References

[1] Yosten GLC Harada CM Haddock C Giancotti LA Kolar GR Patel R GPR160 De-orphanization Reveals Critical Roles in Neuropathic Pain in Rodents. J Clin Invest. (2020) 130:2587–92. https://www.jci.org/articles/view/133270 31999650 10.1172/JCI133270 PMC7190928

[2] Haddock CJ Almeida-Pereira G Stein LM Hayes MR Kolar GR Samson WK Signaling in Rat Brainstem *via* Gpr160 is Required for the Anorexigenic and Antidipsogenic Actions of Cocaine- and Amphetamine-Regulated Transcript Peptide. Am J Physiol Regul Integr Comp Physiol. (2021) 320:R236–R249. 10.1152/ajpregu.00096.2020 33206556 PMC7988768

[3] Vale W Spiess J Rivier C Rivier J. Characterization of a 41-Residue Ovine Hypothalamic Peptide that Stimulates Secretion of Corticotropin and Beta-endorphin. Science (1981) 213:1394–7. 10.1126/science.6267699 6267699

[4] Douglass J McKinzie A Couceyro P. PCR Differential Display Identifies a Rat Brain mRNA that is Transcriptionally Regulated by Cocaine and Amphetamine. J Neurosci (1995) 15(3 II):2471–81. 10.1523/jneurosci.15-03-02471.1995 7891182 PMC6578117

[5] Yosten GLC Haddock CJ Harada CM Almeida-Pereira G Kolar GR Stein LM Past, Present and Future of Cocaine- and Amphetamine-Regulated Transcript Peptide. Physiol Behav (2021) 235:113380. 10.1016/j.physbeh.2021.113380 33705816 PMC8058305

[6] Singh A de Araujo AM Krieger J-P Vergara M Ip CK de Lartigue G. Demystifying Functional Role of Cocaine- and Amphetamine-related Transcript (CART) Peptide in Control of Energy Homeostasis: A Twenty-five Year Expedition. Peptides (2021) 140:170534. 10.1016/j.Peptides.2021.170534 33757831 PMC8369463

[7] Kuhar MJ Job MO. CART Peptide Regulates Psychostimulant-induced Activity and Exhibits a Rate Dependency. J Drug Alcohol Res (2017) 6:1–2. 10.4303/jdar/236032 PMC5718383 29225992

[8] Lau J Herzog H. CART in the Regulation of Appetite and Energy Homeostasis. Front Neurosci (2014) 8:313. 10.3389/fnins.2014.00313 25352770 PMC4195273

[9] Zhang M Han L Xu Y. Roles of Cocaine- and Amphetamine-regulated Transcript in the Central Nervous System. Clin Exp Pharmacol Physiol (2012) 39:586–92. 10.1111/j.1440-1681.2011.05642.x 22077697

[10] Rogge G Jones D Hubert GW Lin Y Kuhar MJ. CART Peptides: Regulators of Body Weight, Reward and other Functions. Nat Rev Neurosci (2008) 9:747–58. 10.1038/nrn2493 18802445 PMC4418456

[11] Fagergren P Hurd Y. CART mRNA Expression in Rat Monkey and Human Brain: Relevance to Cocaine Abuse. Physiol Behav (2007) 92:218–25. 10.1016/j.physbeh.2007.05.027 17631364

[12] Wierup N Sundler F. CART is a Novel Islet Regulatory Peptide. Peptides (2006) 27:2031–6. 10.1016/j.Peptides.2006.02.011 16842887

[13] Ekblad E. CART in the Enteric Nervous System. Peptides (2006) 27:2024–30. 10.1016/j.Peptides.2005.12.015 16759747

[14] Stein J Steiner DF Dey A. Processing of Cocaine- and Amphetamine-regulated Transcript (CART) Precursor Proteins by Prohormone Convertases (PCs) and its Implications. Peptides (2006) 27:1919–25. 10.1016/j.Peptides.2005.10.028 16784796

[15] Koylu EO Balkan B Kuhar MJ Pogun S. Cocaine and Amphetamine Regulated Transcript (CART) and the Stress Response. Peptides (2006) 27:1956–69. 10.1016/j.Peptides.2006.03.032 16822586

[16] Larsen PJ Hunter RG. The Role of CART in Body Weight Homeostasis. Peptides (2006) 27:1981–6. 10.1016/j.Peptides.2005.11.027 16762453

[17] Kuhar MJ. CART Peptides and Drugs of abuse: A review of recent progress. J Drug Alcohol Res (2016) 5:1–6. 10.4303/jdar/235984 PMC5726282 29238623

[18] Moffett M Stanek L Harley J Rogge G Asnicar M Hsiung H Studies of Cocaine- and Amphetamine-regulated Transcript (CART) Knockout mice. Peptides (2006) 27:2037–45. 10.1016/j.Peptides.2006.03.035 16762458

[19] Yermolaieva O Chen J Couceyro PR Hoshi T. Cocaine- and Amphetamine-regulated Transcript Peptide Modulation of Voltage-Gated Ca2+Signaling in Hippocampal Neurons. J Neurosci (2001) 21:7474–80. 10.1523/jneurosci.21-19-07474.2001 11567037 PMC6762924

[20] Lin Y Hall RA Kuhar MJ. CART Peptide Stimulation of G Protein-Mediated Signaling in Differentiated PC12 Cells: Identification of PACAP 6-38 as a CART Receptor Antagonist. NeuroPeptides (2011) 45:351–8. 10.1016/j.npep.2011.07.006 21855138 PMC3170513

[21] Lakatos A Prinster S Vicentic A Hall RA Kuhar MJ. Cocaine- and Amphetamine-regulated Transcript (CART) Peptide Activates the Extracellular Signal-regulated Kinase (ERK) Pathway in AtT20 Cells *via* Putative G-protein Coupled Receptors. Neurosci Lett (2005) 384:198–202. 10.1016/j.neulet.2005.04.072 15908120

[22] Vicentic A Lakatos A Jones D. The CART Receptors: Background and Recent Advances. Peptides (2006) 27:1934–7. 10.1016/j.Peptides.2006.03.031 16713658

[23] Samson WK Salvemini D Yosten GLC. Overcoming Stress, Hunger, and Pain: Cocaine- and Amphetamine-Regulated Transcript Peptide's Promise. Endocrinology (2021) 162:162. 10.1210/endocr/bqab108 PMC8210821 34043767

[24] Abels M Riva M Bennet H Ahlqvist E Dyachok O Nagaraj V CART is Overexpressed in Human Type 2 Diabetic Islets and Inhibits Glucagon Secretion and Increases Insulin Secretion. Diabetologia (2016) 59:1928–37. 10.1007/s00125-016-4020-6 27338624

[25] Brennan DJ O'Connor DP Laursen H Mcgee SF Mccarthy S Zagozdzon R The Cocaine- and Amphetamine-regulated Transcript Mediates Ligand-independent Activation of ERα, and is an Independent Prognostic Factor in Node-negative Breast Cancer. Oncogene (2012) 31:3483–94. 10.1038/onc.2011.519 22139072 PMC5739589

[26] Ahmadian-Moghadam H Sadat-Shirazi M-S Zarrindast M-R. Cocaine- and Amphetamine-regulated Transcript (CART): A Multifaceted NeuroPeptide. Peptides (2018) 110:56–77. 10.1016/j.Peptides.2018.10.008 30391426

[27] Subhedar NK Nakhate KT Upadhya MA Kokare DM. CART in the Brain of Vertebrates: Circuits, Functions and Evolution. Peptides (2014) 54:108–30. 10.1016/j.Peptides.2014.01.004 24468550

[28] Nagelová V Pirník Z Železná B Maletínská L. CART (Cocaine- and Amphetamine-regulated Transcript) Peptide Specific Binding Sites in PC12 Cells have Characteristics of CART Peptide Receptors. Brain Res (2014) 1547:16–24. 10.1016/j.brainres.2013.12.024 24378198

[29] Davenport AP Alexander SPH Sharman JL Pawson AJ Benson HE Monaghan AE International union of Basic and Clinical Pharmacology. LXXXVIII. g Protein-Coupled Receptor List: Recommendations for new Pairings with Cognate Ligands. Pharmacol Rev (2013) 65:967–86. 10.1124/pr.112.007179 23686350 PMC3698937

[30] Meng Q Kim H-C Oh S Lee Y-M Hu Z Oh K-W. Cocaine- and Amphetamine-Regulated Transcript (CART) Peptide Plays Critical Role in Psychostimulant-Induced Depression. Biomolecules Ther (2018) 26:425–31. 10.4062/biomolther.2018.141 PMC6131014 30157614

[31] Thim L Kristensen P Nielsen PF Wulff BS Clausen JT. Tissue-Specific Processing of Cocaine- and Amphetamine-regulated Transcript Peptides in the Rat. Proc Natl Acad Sci (1999) 96:2722–7. 10.1073/PNAS.96.6.2722 10077578 PMC15836

[32] Wierup N Richards WG Bannon AW Kuhar MJ Ahrén B Sundler F. CART knock out mice have Impaired Insulin Secretion and Glucose Intolerance, Altered Beta Cell Morphology and Increased Body Weight. Regul Peptides (2005) 129:203–11. 10.1016/J.REGPEP.2005.02.016 15927717

[33] Miraglia del Giudice E Santoro N Fiumani P Dominguez G Kuhar MJ Perrone L. Adolescents Carrying a Missense Mutation in the CART Gene Exhibit Increased Anxiety and Depression. Depress Anxiety (2006) 23:90–2. 10.1002/DA.20156 16400624

[34] Salinas AG Nguyen CTQ Ahmadi-Tehrani D Morrisett RA. Reduced Ethanol Consumption and Preference in Cocaine- and Amphetamine-regulated Transcript (CART) Knockout Mice. Addict Biol (2014) 19:175–84. 10.1111/J.1369-1600.2012.00475.X 22823101 PMC4148419

[35] Maixnerová J Hlaváček J Blokešová D Kowalczyk W Elbert T Šanda M Structure-Activity Relationship of CART (Cocaine- and Amphetamine-regulated Transcript) Peptide Fragments. Peptides (2007) 28:1945–53. 10.1016/J.PEPTIDES.2007.07.022 17766010

[36] Blechová M Nagelová V Žáková L Demianová Z Železná B Maletínská L. New Analogs of the CART Peptide with Anorexigenic Potency: The Importance of Individual Disulfide Bridges. Peptides (2013) 39:138–44. 10.1016/j.Peptides.2012.09.033 23174349

[37] Maletínská L Maixnerová J Matyšková R Haugvicová R Šloncová E Elbert T Cocaine- and Amphetamine-regulated Transcript (CART) Peptide Specific Binding in Pheochromocytoma Cells PC12. Eur J Pharmacol (2007) 559:109–14. 10.1016/j.ejphar.2006.12.014 17292884

[38] Pražienková V Marek A Maletínská L. Iodination of CART(61‐102) Peptide: Preserved Binding and Anorexigenic Activity in Mice. J Label Compd Radiopharm (2021) 64:61–4. 10.1002/JLCR.3871 32678955

[39] Smith KL Gardiner JV Ward HL Kong WM Murphy KG Martin NM Overexpression of CART in the PVN Increases Food Intake and Weight Gain in Rats. Obesity (Silver Spring) (2008) 16:2239–44. 10.1038/OBY.2008.366 18719668

[40] Asnicar MA Smith DP Yang DD Heiman ML Fox N Chen Y-F Absence of Cocaine- and Amphetamine-regulated Transcript Results in Obesity in Mice Fed a High Caloric Diet. Endocrinology (2001) 142:4394–400. 10.1210/ENDO.142.10.8416 11564703

[41] Vrang N Tang-Christensen M Larsen PJ Kristensen P. Recombinant CART Peptide Induces c-Fos Expression in Central Areas involved in Control of Feeding Behaviour. Brain Res (1999) 818:499–509. 10.1016/S0006-8993(98)01349-3 10082837

[42] Smedh U Scott KA Moran TH. Fourth Ventricular CART Peptide Induces c-fos in the area Postrema and Nucleus of the Solitary Tract *via* a CRF-receptor Dependent Mechanism. Neurosci Lett (2015) 609:124–8. 10.1016/J.NEULET.2015.10.028 26475505

[43] Somalwar AR Choudhary AG Sharma PR B. N Sagarkar S Sakharkar AJ Cocaine- and Amphetamine-regulated Transcript Peptide (CART) Induced Reward Behavior is mediated *via* Gi/o dependent Phosphorylation of PKA/ERK/CREB Pathway. Behav Brain Res (2018) 348:9–21. 10.1016/j.bbr.2018.03.035 29580892

[44] Vicentic A Lakatos A Kuhar MJ. CART (Cocaine- and Amphetamine-regulated Transcript) Peptide Receptors: Specific Binding in AtT20 Cells. Eur J Pharmacol (2005) 528:188–9. 10.1016/J.EJPHAR.2005.11.041 16330022

[45] Kuhar MJ Yoho LL. CART Peptide Analysis by Western Blotting. Synapse (1999) 33:163–71. 10.1002/(sici)1098-2396(19990901)33:3<163:aid-syn1>3.0.co10.1002/(sici)1098-2396(19990901)33:3<163:aid-syn1>3.0.co;2-t2-t 10420164

